# Two pyrrole acids isolated from *Phyllanthus emblica* L. and their bioactivities

**DOI:** 10.1007/s13659-023-00393-0

**Published:** 2023-08-28

**Authors:** Shu-Hui Wang, Cong Guo, Wen-Jin Cui, Qing-Xia Xu, Jun Zhang, Jin-Zhu Jiang, Yan Liu, Sha Chen, Chang Chen, Jin-Tang Cheng, An Liu

**Affiliations:** Institute of Chinese Materia Medica, China Academy of Chinese Medical Sciences, Beijing, 100700 China

**Keywords:** Pyrrole acid, *Phyllanthus emblica* L., Lipid deposition, ATP-binding cassette transporter A1, RAW264.7 macrophages

## Abstract

An undescribed pyrrole acid, 1-(4′-methoxy-4′-oxobutyl)-1* H*-pyrrole-2,5-dicarboxylic acid (**1**) and one known pyrrole acid (**2**) were isolated from the fruits of *Phyllanthus emblica.* The structures of these compounds were elucidated via the comprehensive analyses of IR, HRESIMS, 1D and 2D spectroscopic data. A series of biological assays revealed that compounds **1** and **2** could inhibit LPS-induced over-production of nitric oxide (NO), interleukin-6 (IL-6), monocyte chemotactic protein 1 (MCP-1) and tumor necrosis factor-α (TNF-*α*) by reducing the phosphorylation of extracellular regulated protein kinases (ERK) and c-Jun N-terminal kinases (JNK) in RAW 264.7 cells. Additionally, compounds **1** and **2** were found to reduce lipid deposition and increase the mRNA expression of ATP-binding cassette transporter A1 in oxidized low-density lipoprotein-treated RAW264.7 macrophages.

**Graphical abstract:**

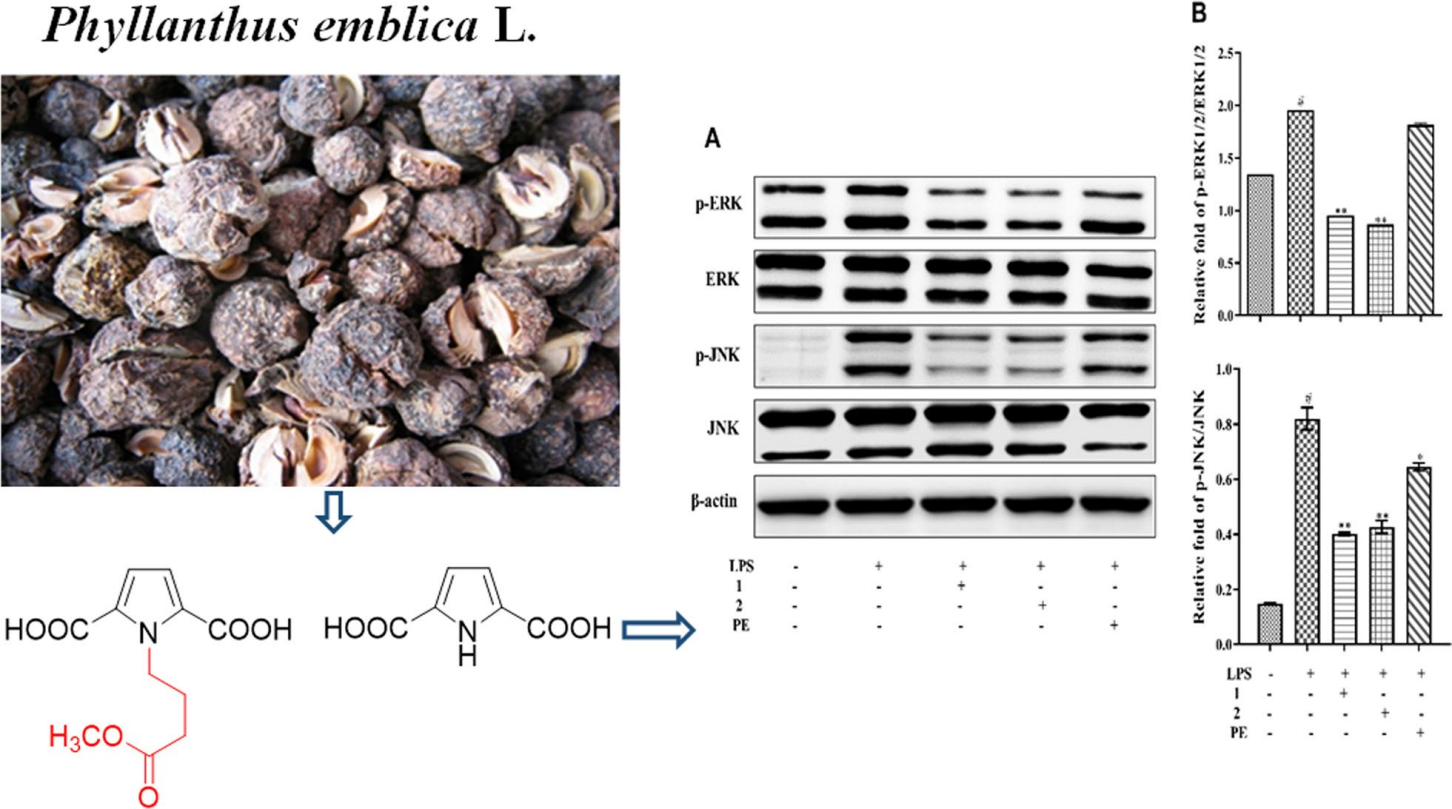

**Supplementary Information:**

The online version contains supplementary material available at 10.1007/s13659-023-00393-0.

## Introduction

Atherosclerosis (AS), a chronic inflammatory disease triggered by the accumulation of cholesterol-containing low-density lipoprotein (LDL) particles on the arterial wall, is the principal contributing factor to many cardiovascular diseases [1]. Oxidized low-density lipoprotein (Ox-LDL), oxidative stress and persistent inflammation are closely associated with the development of AS [2]. Likewise, inflammation markers such as interleukin-6 (IL-6) and tumor necrosis factor (TNF) are independently associated with adverse cardiac outcomes in people with atherosclerosis [3].

Natural products often serve as important lead compounds in medicinal chemistry and so play a highly significant role in the process of drug discovery and development [4]. Traditional medicine practices have often informed the identification of biologically active natural products, such as in the cases of artemisinin and reserpine, which had originally been isolated from traditional Chinese medicine (TCM) preparations and subsequently developed into medicines [5]. *Phyllanthus emblica* L., mainly distributed in subtropical and tropical parts of southeast Asia, has been used for thousands of years in traditional Chinese and Indian medicine for treating diseases such as hemorrhage, diarrhea, jaundice, and dyspepsia etc. [6–9]. Modern research has shown that the *emblica* fruit is a rich source of phenolics, which display a wide range of activities including anti-oxidant [10], anti-viral [11], anti-microbial [12], anti-tumor [13] and anti-bacterial effects [14]. In addition to its medicinal applications, the *emblica* fruit is also highly nutritious, containing high levels of vitamin C, amino acids, and various minerals [15–17]. Accordingly, this fruit has been hailed as “one of the best rejuvenating herbs” [18]. In recent years, as the public has become more health conscious, there has been a flurry of interest in integrating common herbal preparations into both medicinal and dietary regimens as a means of improving human health and preventing disease [19, 20]. Many of the natural products identified in the fruit of *P. emblica*, such as vitamins C and E [21, 22], flavonoids [23], phenolic acid derivatives [24], amino acids, and minerals [25], are either essential human nutrients or have the potential to positively impact overall human health.

**Fig. 1:**
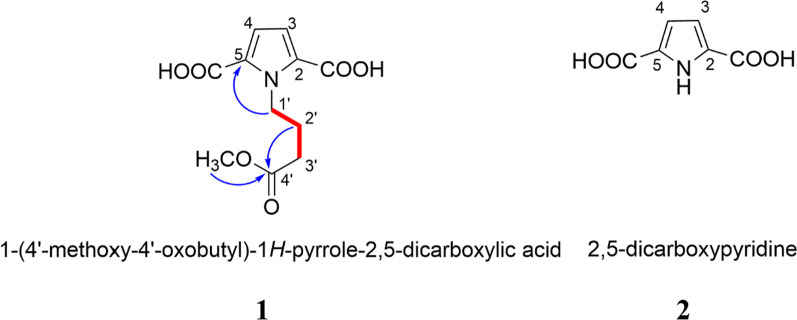
Structures of compounds **1** and **2**. Key ^1^ H-^1^ H COSY (bold bonds in red), HMBC (arrows in blue) correlations of **1**

In our continuing efforts to identify new bioactive natural products and provide a better understanding of the properties of *P. emblica* [26], we initiated a chemical investigation of the fruits of *P. emblica*. This led to the isolation of a previously unidentified pyrrole acid (**1**) and one known analog (**2**) (Fig. 1). Described herein are the isolation and structural elucidation of compounds **1** and **2**, and a study of their inhibitory effects on LPS-induced inflammation and Ox-LDL-induced lipid deposition (Additional file 1).

## Results and discussion

### Structural elucidation

Compound **1** was obtained as colorless crystals. Its molecular formula was C_11_H_13_NO_6_ based on the HRESIMS ion at *m/z* 278.0630 [M + Na] ^+^. The IR absorption bands at 1733 cm^−1^ indicated the presence of ester groups. The ^1^H NMR spectrum (Table 1) showed a COOH singlet at *δ*_H_ 12.25 (2H, s), two olefinic protons [*δ*_H_ 6.83 (2H, s)], three methylene groups [ *δ*_H_ 4.81 (2H, t, *J* = 7.5 Hz), 1.93 (2H, m), 2.20 (2H, t, *J* = 7.5 Hz)], and a methoxy group [*δ*_H_ 3.56 (3H, s)]. The ^13^ C NMR and the DEPT spectra showed 8 carbon signals assigned to one methyl (*δ*_C_ 52.2), three methylene (*δ*_C_ 46.2, 32.0, 28.2), one methine (*δ*_C_ 118.1) and three quaternary carbons (*δ*_C_ 175.3, 163.7, 129.2). These data suggested that **1** possessed a 1*H*-pyrrole-2,5-dicarboxylic acid (**2**) moiety by comparison of these data with those reported in the literature [27]. In addition, detailed examination of the associated HSQC and COSY spectra indicated the presence of a -CH_2_CH_2_CH_2_ (C-1′/C-2′/C-3′) fragment. Furthermore, HMBC cross-peaks of H-2′/C-4′, H-3′/C-4′ and CH_3_/C-4′ established the connection between the -CH_2_CH_2_CH_2_ moiety and a -COOCH_3_ group. The key HMBC correlation from H-1′ to C-2/C-5, together with the above evidence, clearly proved the structure of **1** to be 1-(4′-methoxy-4′-oxobutyl)-1* H*-pyrrole-2,5-dicarboxylic acid, as shown in Fig. 1.

**Table 1:** ^1^ H and ^13^ C NMR spectroscopic date (600 and 150 MHz, DMSO-*d*_*6*_) of compounds **1** and **2**

	1		2	
position	*δ*_H_(*J* in Hz)	*δ*_C_	*δ*_H_(*J* in Hz)	*δ*_C_
2,5		129.2		127.3
3,4	6.83 (s)	118.1	6.83 (s)	114.8
COOH	12.84 (s) for -OH	163.7		162.8
1’	4.81 (t, 7.5)	46.2		
2’	1.93 (m)	28.2		
3’	2.20 (t, 7.5)	32.0		
4’		175.3		
5‘(OCH_3_)	3.54 (s)	52.2		

(δ: ppm)

### Compounds 1 and 2 inhibit LPS-induced production of inflammatory cytokines

**Fig. 2:**
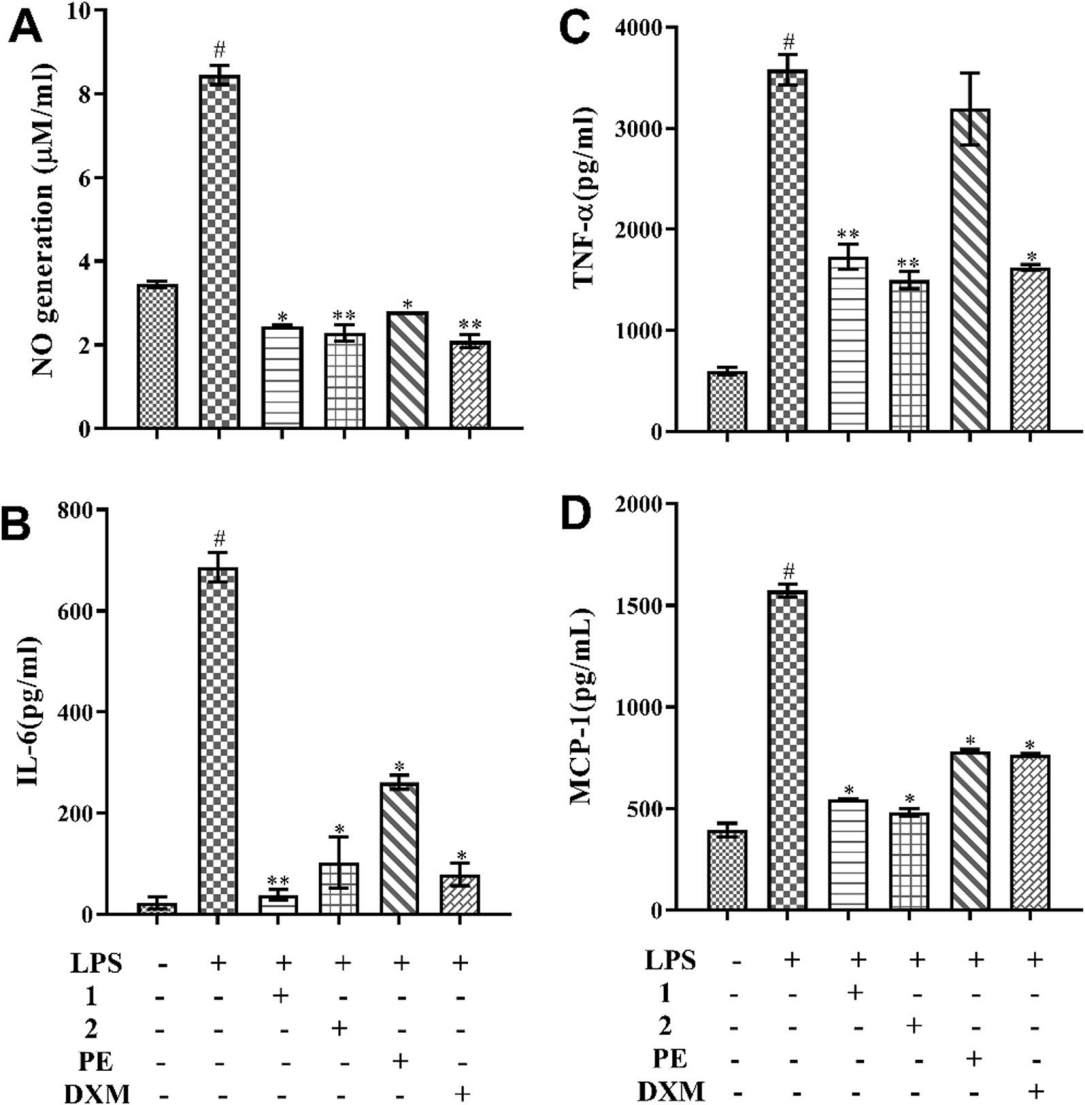
Effect of the two pyrrole acids **1** and **2** and *P. emblica* extract (PE) on LPS-induced NO (**A**), IL-6 (**B**), TNF-α (**C**) and MCP-1 (**D**) production in RAW264.7 cells. Macrophages were pre-treated with/without compounds **1**, **2** (100 *µ*M) or PE (500 *µ*g/mL) for 2 h and then stimulated with 500 ng/mL LPS for 24 h. The production of these markers was measured by Griess/ELISA. Dexamethasone (DXM) was set as a control (100 *µ*M). Control was cultured without samples and LPS. The data presented are the means ± SD, #*p < 0.05* significant compared with the control group, and *p < 0.05, **p < 0.01 significant compared with LPS-treated group

As illustrated in Fig. 2A, compounds **1**, **2** and the crude extract of *P. emblica* (PE) inhibited NO production from LPS-treated RAW264.7 cells (*p < 0.05*). To determine the anti-inflammatory activity of the isolates (**1** and **2**) and PE, we investigated their effects on LPS-induced IL-6, TNF-*α* and MCP-1 production in RAW264.7 cells. Compared with a control group, the production of IL-6, TNF-*α* and MCP-1 was markedly increased (*p < 0.05*) after stimulation with LPS, indicating the validity of our model system. As shown in Fig. 2B‒D, PE was capable of reducing the release of IL-6 and MCP-1 (*p < 0.05*). However, it showed no effects on TNF-*α* secretion. Contrastingly, compound **1** could significantly inhibit the release of all three inflammatory cytokines, wherein the inhibition of TNF-*α* and IL-6 was pronounced (*p < 0.01*), and the inhibition of MCP-1 significant (*p < 0.05*). Compound **2** exhibited strong inhibition of TNF-*α* secretion (*p < 0.01*) as well as inhibition of the production of IL-6 and MCP-1 (*p < 0.05*). The cell viability at 50 *µ*M and at 100 *µ*M of compounds **1** and **2** was evaluated using an MTT assay in RAW264.7 cells. Results showed that the survival rate of the cells was above 78% at both concentrations, indicating that compounds **1** and **2** exhibited weak cytotoxicity to RAW 264.7 cells.

### Compounds 1 and 2 inhibit LPS-induced phosphorylation of ERK and JNK

**Fig. 3:**
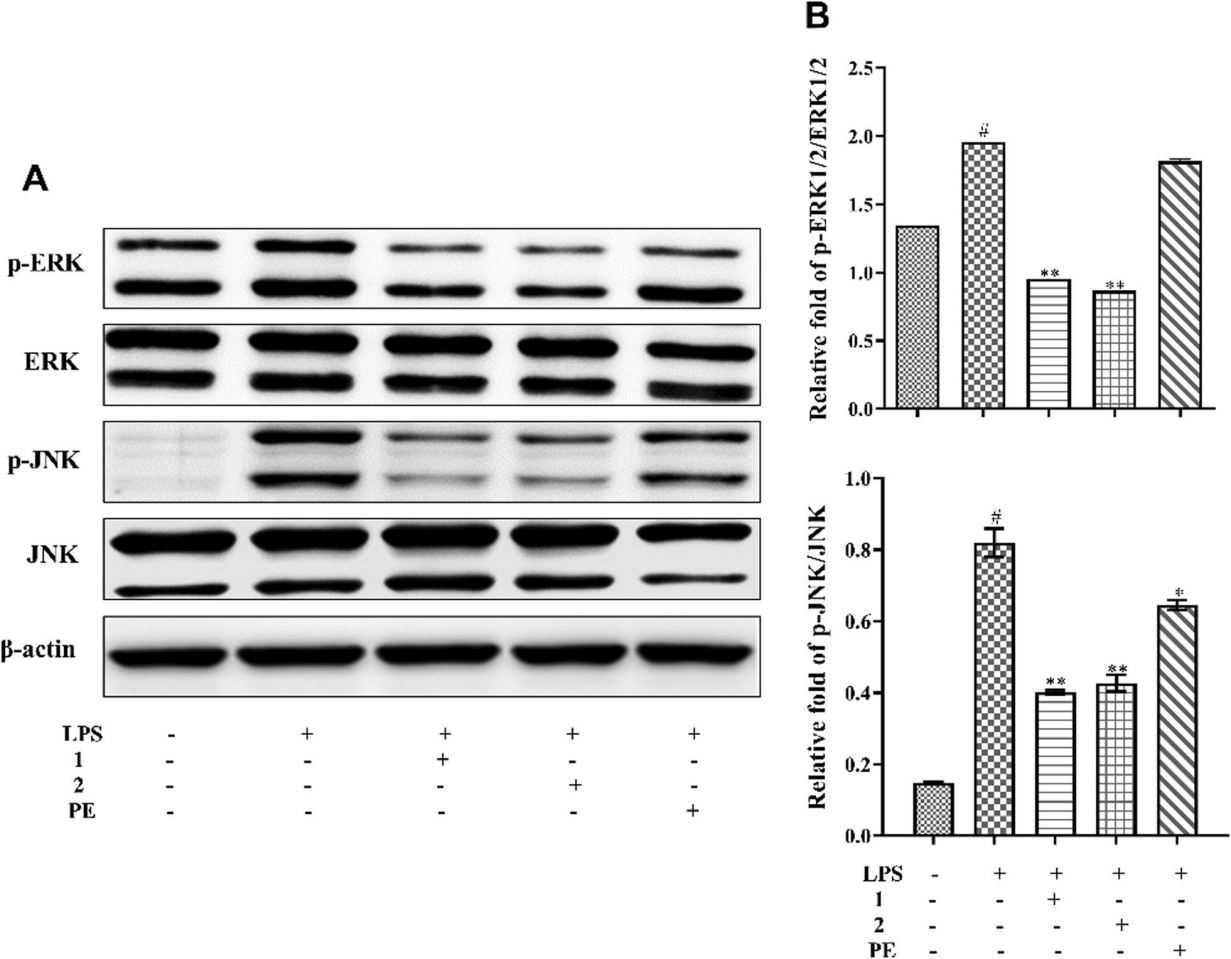
Effects of the two pyrrole acids **1** and **2** and PE on the LPS-induced phosphorylation of the MAPK signaling pathway in RAW264.7 macrophages. Cells were pretreated with/without compounds **1**, **2** (100 *µ*M) or PE (500 *µ*g/mL) for 2 h and then stimulated with LPS (500 ng/mL) for 24 h. **A**–**B**) Whole cell lysates were analyzed by western blotting for detection of phosphorylated p-ERK, ERK, p-JNK, and JNK proteins. Control was cultured without samples and LPS. The data presented are the means ± SD, #*p < 0.05* significant compared with the control group, and *p < 0.05, **p < 0.01

Accumulating evidence suggests that the mitogen-activated protein kinases (MAPK) pathway serves as one of the most important intracellular signaling cascades in pro-inflammatory responses [28]. To investigate whether the interaction of either of these two pyrrole acids or the PE with proteins related to the MAPK signaling pathway was impacting inflammatory effects, we examined the effects of compounds **1**, **2** and PE on the LPS-stimulated phosphorylation of ERK and JNK in RAW264.7 cells using western blot analysis. As shown in Fig. 3, LPS significantly increased the phosphorylation of ERK and JNK (*p < 0.05*), compared with the normal control group. In contrast, pretreatment with compounds **1** and **2** dramatically suppressed the phosphorylation of ERK and JNK (*p < 0.01*). PE only inhibited the phosphorylation of JNK (*p < 0.05*), but had no effect on ERK phosphorylation. Hence, we speculate that the anti-inflammatory effects of *P. emblica* may be related to the ability of these two pyrrole acids to inhibit the phosphorylation of JNK and ERK proteins in the MAPK pathway.

### Compounds 1 and 2 attenuate Ox-LDL-induced lipid deposition

**Fig. 4:**
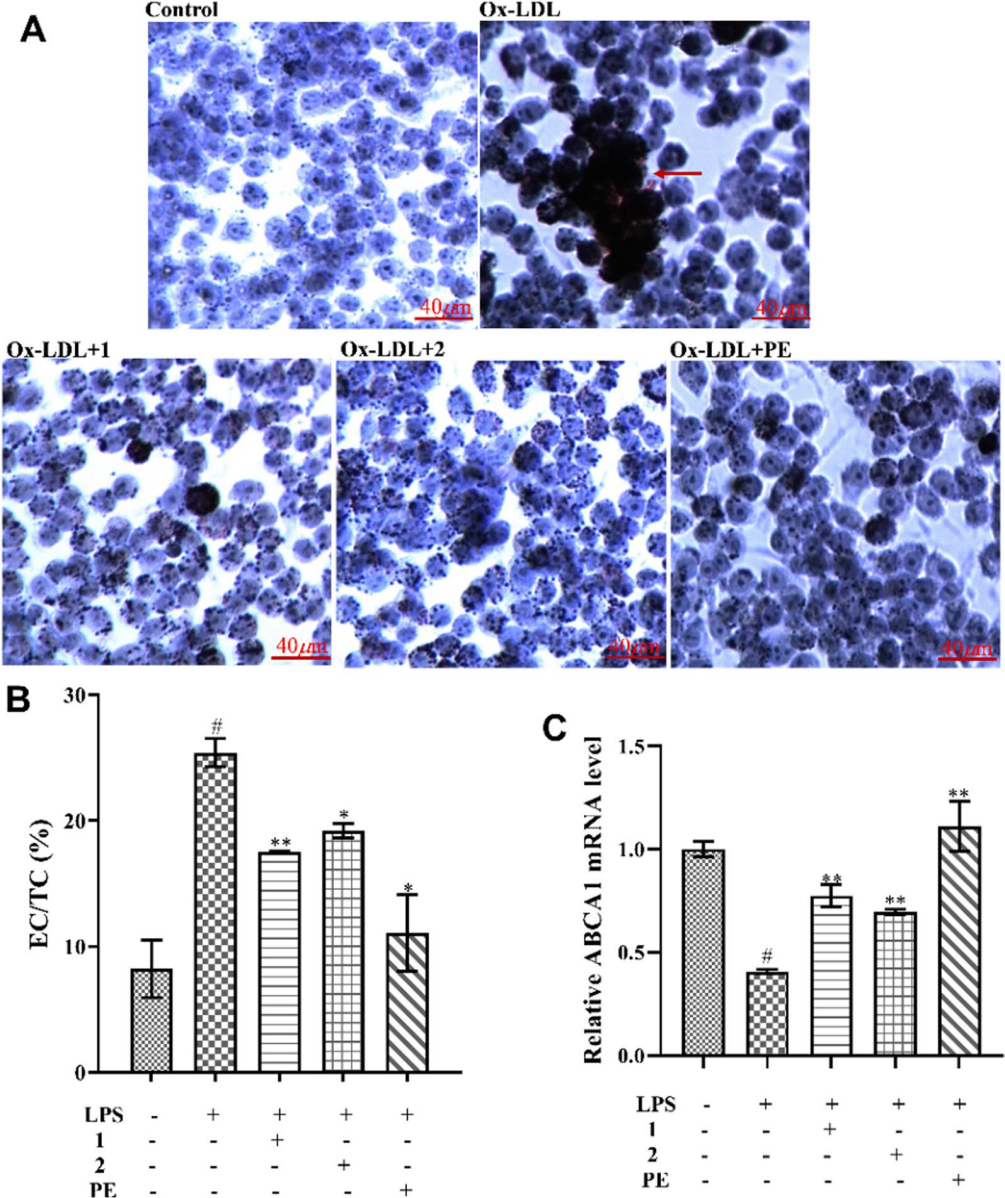
Effects of two pyrrole acids **1** and **2** and PE on lipid accumulation. (A) RAW264.7 cells were treated with ox-LDL (50 *µ*g/mL) in the presence or absence of compounds **1**, **2** (100 *µ*M) or PE (500 *µ*g/mL). (B) After treatment, cultured cells were treated with Ox-LDL (50 *µ*g/mL) for an additional 24 h. Then, macrophages were lysed and the intracellular TC content and FC content were quantified according to the protocol by the manufacturers. (C) After treatment, cultured cells were treated with Ox-LDL (50 *µ*g/mL) for an additional 24 h. The mRNA expression of ABCA1 was assessed by real-time PCR, and expressed as fold induction relative to control. The date presented are the means ± SD, #*p < 0.05* significant compared with the control group, and *p < 0.05, **p < 0.01

Given the relationship between the accumulation of foam cells and the formation of arterial fatty streaks, it has been reported that certain products that inhibit foam cell formation may prevent the progression of atherosclerosis [29, 30]. To investigate the effect of these two pyrrole acids and PE on Ox-LDL-induced lipid accumulation in macrophage-derived foam cells, RAW264.7 cells were pretreated with either of the two pyrrole acids or PE and then incubated with 50 *µ*g/mL Ox-LDL to induce foam cell formation. Confirmation of foam cell formation was confirmed by oil red O staining. As shown in Fig. 4A, Ox-LDL-stimulated cells exhibited more lipid droplets than the normal control cells, suggesting that Ox-LDL significantly promoted foam cell formation. Those cells that had been treated with compounds **1**, **2** and PE, exhibited less staining with oil red O stain.

The generation of foam cells is related to the imbalance of cholesterol influx, esterification and efflux. Therefore, cellular cholesterol levels were quantified. It was found that the CE/TC ratio was dramatically increased in Ox-LDL-treated cells (*p < 0.05*). Moreover, compounds **1**, **2** and PE were all capable of attenuating the CE/TC elevation observed in Ox-LDL-treated cells (*p < 0.01*, Fig. 4B).

The ATP-binding cassette transporter A1 (ABCA1) regulates lipid deposition by acting on the reverse cholesterol transport pathway [31]. Therefore, we examined the effects of compounds **1**, **2** and PE on the expression of ABCA1 mRNA by RT-PCR. The results showed that compounds **1**, **2** and PE upregulated the mRNA expression of ABCA1 in Ox-LDL-treated RAW 264.7 cells (Fig. 4C).

## Materials and methods

### General experimental procedure

IR spectra were recorded on a JASCO FT/IR-480 spectrophotometer and reported as wave number (cm^−1^). ^1^ H, ^13^ C NMR spectra and 2D NMR spectra were recorded on a Bruker Avance III 600 spectrometer. Chemical shifts (*δ*) were expressed in ppm with reference to TMS. HRESIMS data were obtained on an Agilent UPLC/TOF-MS spectrometer. Column chromatography (CC) was performed on silica gel (200‒300 *µ*m; Qingdao Marine Chemical Co. Ltd., China), RP-18 silica gel (SMB-100-25/45, FUJI, Japan) and Sephadex LH-20 (GE Healthcare Bio-Sciences AB SE-75,184 Uppsala, Sweden). Preparative HPLC was performed on Agilent 1100 apparatus equipped with a YMC-Pack ODS-A column (12 *µ*m, 10 × 25 mm). Biological assay data were recorded on microplate reader (Thermo Multiskan PC).

### Plant materials

The dried fruits of *P. emblica* at the commercially mature stage were purchased from Beijing Herborist Chinese Herbal Pieces Co., Ltd., and identified by Dr. Wei Sun (Institute of Chinese Materia Medica, China Academy of Chinese Medical Sciences). A voucher specimen (201,608 M) was stored in the herbarium at the department of medicinal plants, Institute of Chinese Materia Medica, China Academy of Chinese Medical Sciences (Beijing 100,700, China).

### Extraction and isolation

The dried fruits of *P. emblica* (17 kg) were grounded into powder using a disintegrator, and then extracted two times with 50% ethanol aqueous (85 L) at room temperature for 24 h. After filtration and evaporation in vacuo, a gummy residue (5.78 kg) was obtained, which was taken up in H_2_O (7 L) and successively partitioned with petroleum ether, dichloromethane, ethyl acetate and *n*-butanol 2 times (3 L each time). The ethyl acetate fraction (750.9 g) was absorbed on silica gel and subjected to column chromatography (CH_2_Cl_2_/ MeOH as eluting gradients) to give 5 main fractions (Fr.1‒Fr.5) based on TLC analysis. Fr.2 (589.6 g) was further subjected to column chromatography (silica gel; CH_2_Cl_2_/EtOAc step gradients) to give 9 subfractions by TLC analysis. Fr.2.3 (10 g) was further absorbed on silica gel and subjected to column chromatography (petroleum ether/EtOAc step gradients) to give 11 subfractions. Fr.2.3.7 (507 mg) was first subjected to Sephadex LH20 and then recrystallization to give compound **1** (60 mg). Fr.2.4 (7 g) was submitted to ODS eluting with an aqueous methanol gradient to afford 9 portions by TLC analysis. Fr.2.4.5 (124 mg) in methanol and the filtrate were further separated by semi-preparative HPLC (MeOH/H_2_O, 25%) to yield compound **2** (81 mg).

1-(4′-methoxy-4′-oxobutyl)-1* H*-pyrrole-2,5-dicarboxylic acid (**1)**: colorless crystals (MeOH); mp: 180‒181 °C; UV(MeOH) *λ*_*max*_ (log *ε*) 276 (1.274), 211 (0.704) nm; IR (KBr) *v*_*max*_ 2957, 1733, 1703, 1668, 1516, 1416, 1260, 1190, 777 cm^−1^; ^1^ H and ^13^ C NMR spectroscopic date, Table 1; HRESIMS *m/z* 278.0630 [M + Na] ^+^ (calc’d for C_11_H_13_NO_6,_ 278.0635).

### Cell culture

The RAW 264.7 cell line was purchased from the School of Basic Medicine of Peking Union Medical College (Beijing, China). Cells were incubated in Dulbeco’s modified Eagle’s medium (DMEM) supplemented with 10% fetal bovine serum (FBS), 2 mM glutamine, 100 units/mL penicillin and streptomycin at 37 ^o^C in a humidified atmosphere of 5% CO_2_. According to experiments, cells were randomly divided into three groups, including control (vehicle DMSO); LPS/Ox-LDL treated; LPS/Ox-LDL and compounds **1**/**2**/PE treated. After 24 h of incubation, cells were subjected to compounds **1**, **2** and PE extract respectively for 2 h followed by treatment with LPS (500 ng/mL) or Ox-LDL (50 *µ*g/mL) for 24 h. Subsequently, cells were harvested and extracted for further analysis.

### Cell viability assay

After incubated in 96-well culture plates (1 × 10^5^ cells/mL) for 24 h, RAW 264.7 cells were treated with compounds **1** and **2** at different concentrations (50 *µ*M and 100 *µ*M)) respectively for 24 h. Then, RAW 264.7 cells were incubated in 0.5 mg/mL MTT at 37 °C for 4 h. The MTT-containing supernatant was removed after centrifugation. Each well was treated with DMSO (100 *µ*L) at 37 ℃ for 10 min. Subsequently, absorbance values were measured at 450 nm using a microplate reader (Thermo Multiskan PC, America).

### Inflammatory cytokine analysis

Cells were administrated with compounds **1**, **2** (100 *µ*M) or PE (500 *µ*g/mL) for 2 h, and then treated with LPS (500 ng/mL) for 24 h. The culture medium was separated and collected the supernatant. NO concentration in the supernatant was measured by Griess assay. The absorbance of NO concentration was determined by a microplate reader at 540 nm. The content of IL-6, TNF-*α* and MCP-1 levels in the supernatant was quantified by enzyme linked immunosorbent assay (ELISA) kits (BD OptEIA™, USA; R&D systems, Minneapolis, USA) according to the manufacturer’s protocols.

### Western blot analysis

After cells were treated with compounds **1**, **2** (100 *µ*M) or PE (500 *µ*g/mL) for 2 h, the cells were stimulated by LPS (500 ng/mL) for 24 h. Then, cells were washed twice by using ice-cold phosphate buffer saline (PBS, PH7.4) and lysed in whole-cell lysis buffer on ice to yield the cellular proteins. The protein concentration was quantified according to the bicinchoninic acid (BCA) method. Aliquots containing 20 *µ*g of protein were separated on 10% sodium dodecyl sulfate polyacrylamide gel electrophoresis (SDS-PAGE) and electroblotted onto polyvinylidene difluoride membranes (PVDF, Millipore, Billerica, MA, USA). The membranes were blocked with 5% skim milk in Tri-buffered saline tween (TBST) for 1 h at room temperature. To detect the target proteins, the PVDF membranes were incubated with primary antibodies that were specific to the phospho-extracellular regulated protein kinases, extracellular regulated protein kinases, phospho-c-Jun *N*-terminal kinase, c-Jun *N*-terminal kinase and *β-*actin (p-ERK, ERK, p-JNK, JNK, *β-*actin; 1:1000; Cell signaling Technology In, Beverly, MA) overnight at 4 °C. Sequentially, the membranes were rinsed three times with TBST incubated for 1 h with horseradish peroxidase-conjugated secondary antibody (1:5000, Cell signaling Technology In, Beverly, MA). After subsequent washes with TBST and deionized water, membrane bound antibodies were detected using a chemiluminescence (ECL) system (Thermo, USA) and band intensities were quantified using imaging systems analysis software. (Tanon4800, Shanghai, China).

### Oil red O staining

RAW264.7 cells, which had been pre-treated with compounds **1**, **2** (100 *µ*M) and PE (500 *µ*g/mL) for 2 h previously, were stimulated by Ox-LDL (50 *µ*g/mL) for 24 h. After being rinsed once with PBS, the cells were fixed with 10% (v/v) paraformaldehyde for 15 min. Then, cells were washed with double-distilled water (ddH_2_O) and stained with filter 0.5% Oil Red O solution at room temperature for another 15 min. Subsequently, Mayer’s hematoxylin was added to restrain the nucleus for 2 min. Finally, after further washing the cells with ddH_2_O to remove the background staining, the cells were photographed randomly under the microscope.

### Cellular cholesterol quantification

Cells, which had been previously treated with compounds **1**, **2** (100 *µ*M) or PE (500 *µ*g/mL) for 2 h, were stimulated by Ox-LDL (50*µ*g/mL) for 24 h. Then, cells were extracted with chloroform: isopropanol: IGEPAL CA-630 (7:11:0.1) in a micro homogenizer. Cells were centrifuged to remove insoluble material. The organic phase was transferred and dried at 50 °C to remove chloroform, before being dissolved dried lipids with the cholesterol assay buffer. The content of cellular total cholesterol (TC) and free cholesterol (FC) was determined using a cholesteryl assay kit (Sigma-Aldrich, MAK043). The cholesteryl ester (CE) was calculated as follow: CE = TC–FC.

### Real-time polymerase chain reaction

Cells, which had been previously treated with compounds **1**, **2** (100 *µ*M) or PE (500 *µ*g/mL) for 2 h, were stimulated by Ox-LDL (50*µ*g/mL) for 24 h. According to the manufacturer’s instructions, total RNA was isolated using TRIzol reagent (Invitrogen Co., Carlsbad, CA, USA). The concentration of RNA was measured by a spectrophotometer, and the cells with an OD value (A260/A280) between 1.8-2.0 were reverse transcribed into cDNA using the synthesis kits (iScript™ cDNA Synthesis Kit; Bio-Rad). Quantitative RT-PCR was performed using a QuantiFast SYBR® Green PCR Kit (Roche) and ABI PRISM 7300 PCR Machine (Applied Biosystems). The cDNA was amplified at 90°C for 10 min, 40 cycles at 95°C for 15 s, 60°C for 60 s. Final extension was performed for 7 min at 72°C. The PCR products were electrophoresed on 2% agarose gel and stained with ethidium bromide. PCR primers were as follows: ABCA1, 5’-GCAGATCA AGCATCCCAAC T-3’ (forward) and 5’-CCAGAGAATGT TTCATTGTCCA-3’ (reverse).

### Statistical analysis

The results are expressed as a mean ± standard deviation (SD) from at least three independent experiments. Statistical elaborations were performed on GraphPad Prism 7 (CA, USA). For the statistical comparison of means difference between the samples, the Student’s *t*-test were used by SPSS20 (IBM, USA), respectively. The results were considered as significantly different at *p < 0.05* and that of *p < 0.01* was taken to be extremely significant.

## Conclusion

In conclusion, two pyrrole acids (**1** and **2**) were isolated and characterized from the fruits of *P. emblica*. These compounds were identified as 1-(4-methoxy-4-oxobutyl)-1* H*-pyrrole-2,5-dicarboxylic acid and 1* H*-pyrrole-2,5-dicarboxylic acid through various spectroscopic data. Pharmacological experiments showed that compounds **1** and **2** inhibited the production of LPS-induced NO, IL-6, TNF-*α* and MCP-1 in RAW264.7 cells, and that these effects were mediated by the ERK and JNK pathway. Moreover, compounds **1** and **2** effectively reduced lipid deposition induced by Ox-LDL in macrophages. These findings indicate that compounds **1** and **2** may be effective in inhibiting the progression of AS and have potential for the prevention and treatment of AS.

### Supplementary Information

**Additional file 1. **Additional information.

## References

[1] Bäck M, Hansson GK (2015). Anti-inflammatory therapies for atherosclerosis. Nat Rev Cardiol.

[2] Liu Z, Xu S, Huang X, Wang J, Gao S, Li H, Zhou C, Ye J, Chen S, Jin ZG, Liu P (2015). Cryptotanshinone, an orally bioactive herbal compound from Danshen, attenuates atherosclerosis in apolipoprotein E-deficient mice: role of lectin-like oxidized LDL receptor-1 (LOX-1). Br J Pharm.

[3] Angelovich TA, Hearps AC, Jaworowski A (2015). Inflammation-induced foam cell formation in chronic inflammatory disease. Immunol Cell Biol.

[4] Newman DJ, Cragg GM (2020). Natural products as sources of new drugs over the nearly four decades from 01/1981 to 09/2019. J Nat Prod.

[5] Cragg GM, Newman DJ (2013). Natural products: a continuing source of novel drug leads. Biochim Biophys Acta.

[6] Kumaran A, Karunakaran RJ (2006). Nitric oxide radical scavenging active components from Phyllanthus emblica L. Plant Food Hum Nutr.

[7] Luo W, Zhao M, Yang B, Ren J, Shen G, Rao G (2011). Antioxidant and antiproliferative capacities of phenolics purified from *Phyllanthus emblica* L. fruit. Food Chem.

[8] Mathai RT, Tonse R, Kalekhan F, Colin MD, Prabhu HS, Rao S, Baliga MS, Watson RR (2015). Chapter 3-Amla in the prevention of aging: scientific validation of the ethnomedicinal claims. Foods and Dietary supplements in the Prevention and Treatment of Disease in older adults.

[9] Yang B, Kortesniemi M, Liu P, Karonen M, Salminen JP (2012). Analysis of hydrolyzable tannins and other phenolic compounds in emblic leafflower (*Phyllanthus emblica* L.) fruits by high performance liquid chromatography-electrospray lonization mass spectrometry. J Agric Food Chem.

[10] Sreeramulu D, Raghunath M (2010). Antioxidant activity and phenolic content of roots, tubers and vegetables commonly consumed in India. Food Res Int.

[11] Ryu YB, Kim JH, Park SJ, Chang JS, Rho MC, Bae KH, Park KH, Lee WS (2010). Inhibition of neuraminidase activity by polyphenol compounds isolated from the roots of *Glycyrrhiza uralensis*. Bioorg Med Chem Lett.

[12] Buzzini P, Arapitsas P, Goretti M, Branda E, Turchetti B, Pinelli P, Ieri F, Romani A (2008). Antimicrobial and antiviral activity of hydrolysable tannins. Mini-rev Med Chem.

[13] Adams LS, Seeram NP, Aggarwal BB, Takada Y, Sand D, Heber D (2006). Pomegranate juice, total pomegranate ellagitannins, and punicalagin suppress inflammatory cell signaling in colon cancer cells. J Agric Food Chem.

[14] Matito C, Mastorakou F, Centelles J, Torres J, Cascante M (2003). Antiproliferative effect of antioxidant polyphenols from grape in murine Hepa-1C1C7. Eur J Nutr.

[15] Gao Q, Xiang HY, Chen W, Ling J, Chen X, Li J, Zhang TD, Li XM, Zou M, Yang GY, Hu QF (2019). Two new isobenzofuranone derivatives from *Phyllanthus emblica* and their bioactivity. Chem Nat Comp.

[16] Mahata S, Pandey A, Shukla S, Tyagi A, Husain SA, Das BC, Bharti AC (2013). Anticancer activity of *Phyllanthus emblica* Linn. (indian gooseberry): inhibition of transcription factor AP-1 and HPV gene expression in cervical cancer cells. Nutr Cancer.

[17] Yokozawa T, Kim HY, Kim HJ, Tanaka T, Sugino H, Okubo T, Chu DC, Juneja LR (2007). Amla (*Emblica officinalis* Gaertn.) Attenuates age-related renal dysfunction by oxidative stress. J Agric Food Chem.

[18] Majeed M, Bhat B, Jadhav AN, Srivastava JS, Nagabhushanam K (2009). Ascorbic acid and tannins from *Emblica* officinalis Gaertn. fruits-A revisit. J Agric Food Chem.

[19] Liu H, Qiu N, Ding H, Yao R (2008). Polyphenols contents and antioxidant capacity of 68 chinese herbals suitable for medical or food uses. Food Res Int.

[20] Yang B, Liu P (2014). Composition and biological activities of hydrolysable tannins of fruits of *Phyllanthus emblica*. J Agric Food Chem.

[21] Colucci R, Dragoni F, Conti R, Pisaneschi L, Lazzeri L, Moretti S (2015). Evaluation of an oral supplement containing *Phyllanthus emblica* fruit extracts, vitamin E, and carotenoids in vitiligo treatment. Dermatol Ther.

[22] Scartezzini P, Antognoni F, Raggi MA, Poli F, Sabbioni C (2006). Vitamin C content and antioxidant activity of the fruit and of the ayurvedic preparation of *Emblica officinalis* Gaertn. J Ethnopharmacol.

[23] Ruangchakpet A, Sajjaanantakul T (2007). Effect of browning on total phenolic, flavonoid content and antioxidant activity in indian gooseberry (*Phyllanthus emblica* Linn). Kasetsart J (Nat Sci).

[24] Rose K, Wan C, Thomas A, Seeram NP, Ma H (2018). Phenolic compounds isolated and identified from Amla (*Phyllanthus emblica*) juice powder and their antioxidant and neuroprotective activities. Nat Prod Commun.

[25] Gaire BP, Subedi L (2014). Phytochemistry, pharmacology and medicinal properties of *Phyllanthus emblica* Linn. Chin J Integr Med..

[26] Wang SH, Cheng JT, Guo C, Cui WJ, Shi J, Liu A (2019). Study on chemical constituents of. Phyllanthus emblica Zhongcaoyao.

[27] Košt̀álová D, Hrochová V, Suchý V, Buděšínský M, Ubik K (1992). Two pyrrole acids from *Berberis koreana*. Phytochemistry.

[28] Gaestel M, Kotlyarov A, Kracht M (2009). Targeting innate immunity protein kinase signaling in inflammation. Nat Rev Drug Discov.

[29] Ma Q, Zhang XM, Jiang JG, Zhu W (2017). Apigenin-7-O-β-D-glucuronide inhibits modified low-density lipoprotein uptake and foam cell formation in macrophages. J Funct Foods.

[30] Shen CY, Jiang JG, Huang CL, Zhu W (2018). Gypenoside LVI attenuates foam cell formation by promoting cholesterol export and inhibiting inflammation response. J Funct Foods.

[31] Shrestha E, Hussein MA, Savas JN, Ouimet M, Barrett TJ, Leone S, Yates JR, Moore KJ, Fisher EA, Garabedian MJ (2016). Poly (ADP-ribose) polymerase 1 represses liver x receptor-mediated ABCA1 expression and cholesterol efflux in macrophages. J Biol Chem.

