# Supplementary material for: Two pyrrole acids isolated from Phyllanthus emblica L. and their bioactivities
Source: Nat Prod Bioprospect. 2023 Aug 28;13(1):26. doi: 10.1007/s13659-023-00393-0 (PMC10462542; doi:10.1007/s13659-023-00393-0)
Supplement: Supplementary file 1 — Additional file 1. Additional information. [file 13659_2023_393_MOESM1_ESM.docx]

**Supporting Information**

**Two pyrrole acids isolated from *Phyllanthus emblica* L*.* and their bioactivities**

Shu-Hui Wang^‡^, Cong Guo^‡^, Wen-Jin Cui, Qing-Xia Xu, Jun Zhang, Jin-Zhu Jiang, Yan Liu, Sha Chen, Chang Chen, Jin-Tang Cheng*, An Liu*

Institute of Chinese Materia Medica, China Academy of Chinese Medical Sciences, Beijing 100700, China

^‡^ S.-H. Wang, C. Guo contributed equally.

^*^ Corresponding author. Tel.: +86-10-6401-4411 (ext. 2848); Fax: +86-10-6401-3996;

E-mail address: [jtcheng@icmm.ac.cn,](mailto:jtcheng@icmm.ac.cn,) [la62@163.com](mailto:la62@163.com)

Contents of Additional file 1

Figure S1. ^1^H NMR (CD_3_OD) spectrum of compound **1^………………………………^**3

Figure S2. ^1^H NMR (DMSO-*d*_6_) spectrum of compound **1^………………………………^**3

Figure S3. ^13^C NMR spectrum of compound **1^………………………………^**4

Figure S4. HSQC spectrum of compound **1^………………………………^**5

Figure S5. COSY spectrum of compound **1^………………………………^**6

Figure S6. HMBC spectrum of compound **1^………………………………^**7

Figure S7. ^1^H NMR spectrum of compound **2^………………………………^**8

Figure S8. ^13^C NMR spectrum of compound **2^………………………………^**9

Figure S9. HRESIMS of compound **1^………………………………^**10

Figure S10. IR spectrum of compound **1^………………………………^**10

Figure S11. Effects of compounds **1** and **2** on cell cytotoxicity**^………………………………^**11


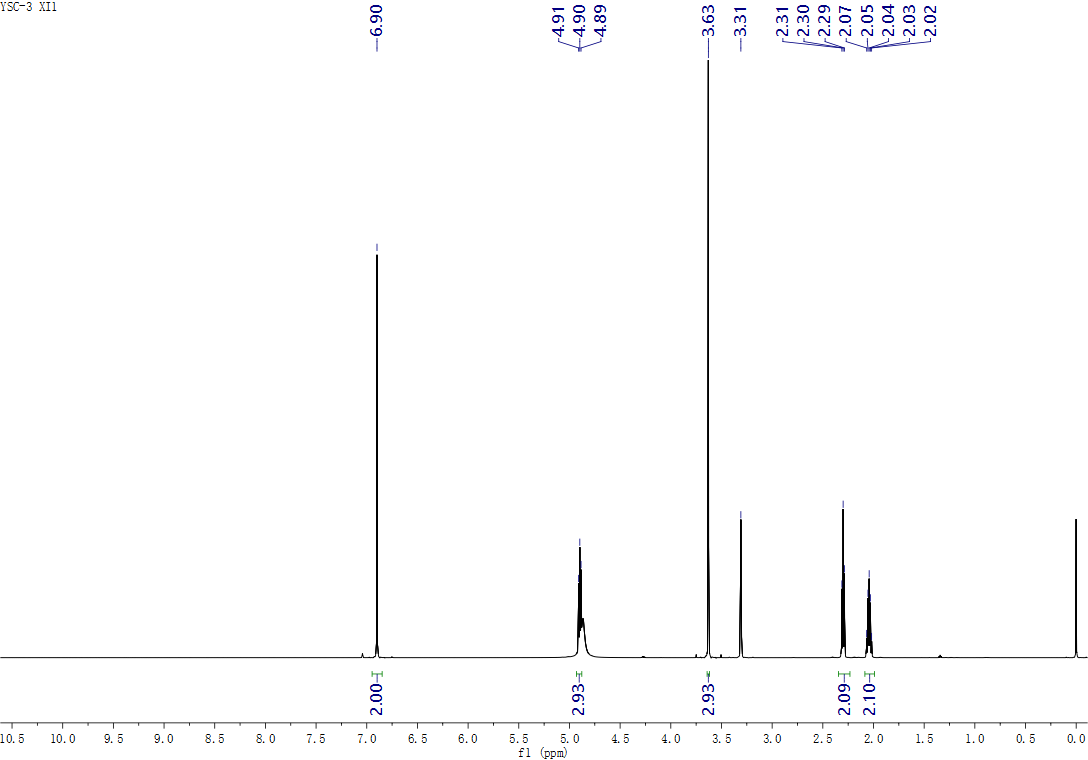


**Figure S1. ^1^H NMR (CD_3_OD) spectrum of compound 1**


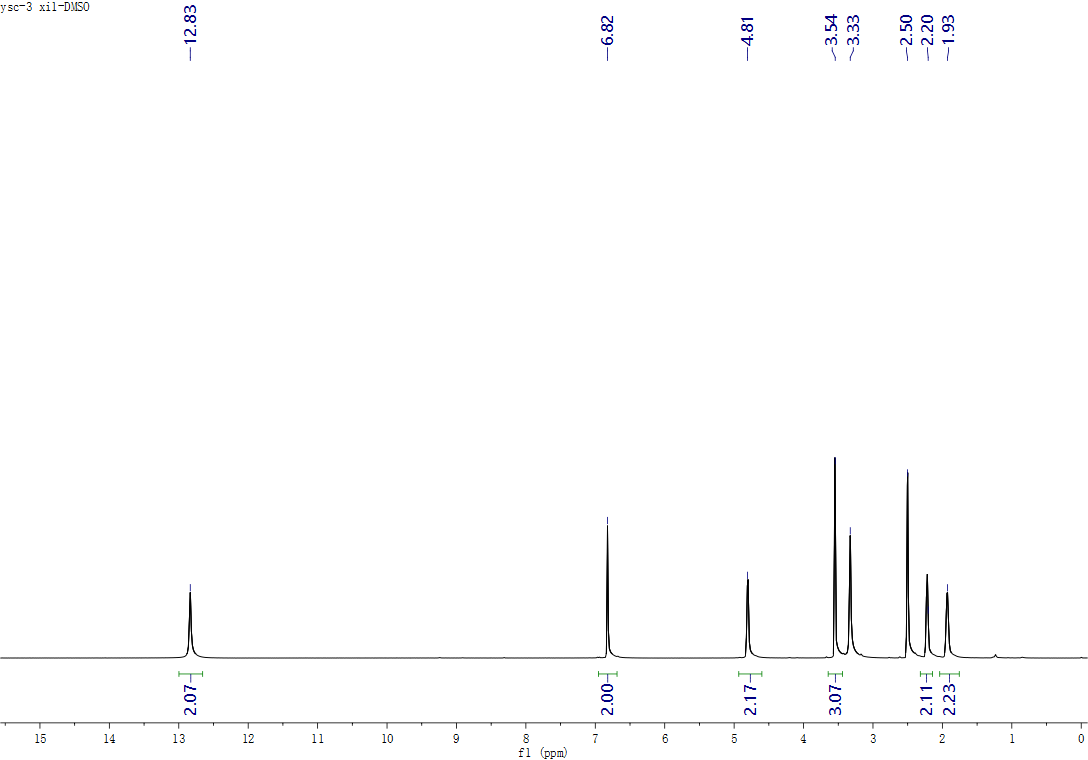


**Figure S2. ^1^H NMR (DMSO-*d_6_*) spectrum of compound 1**


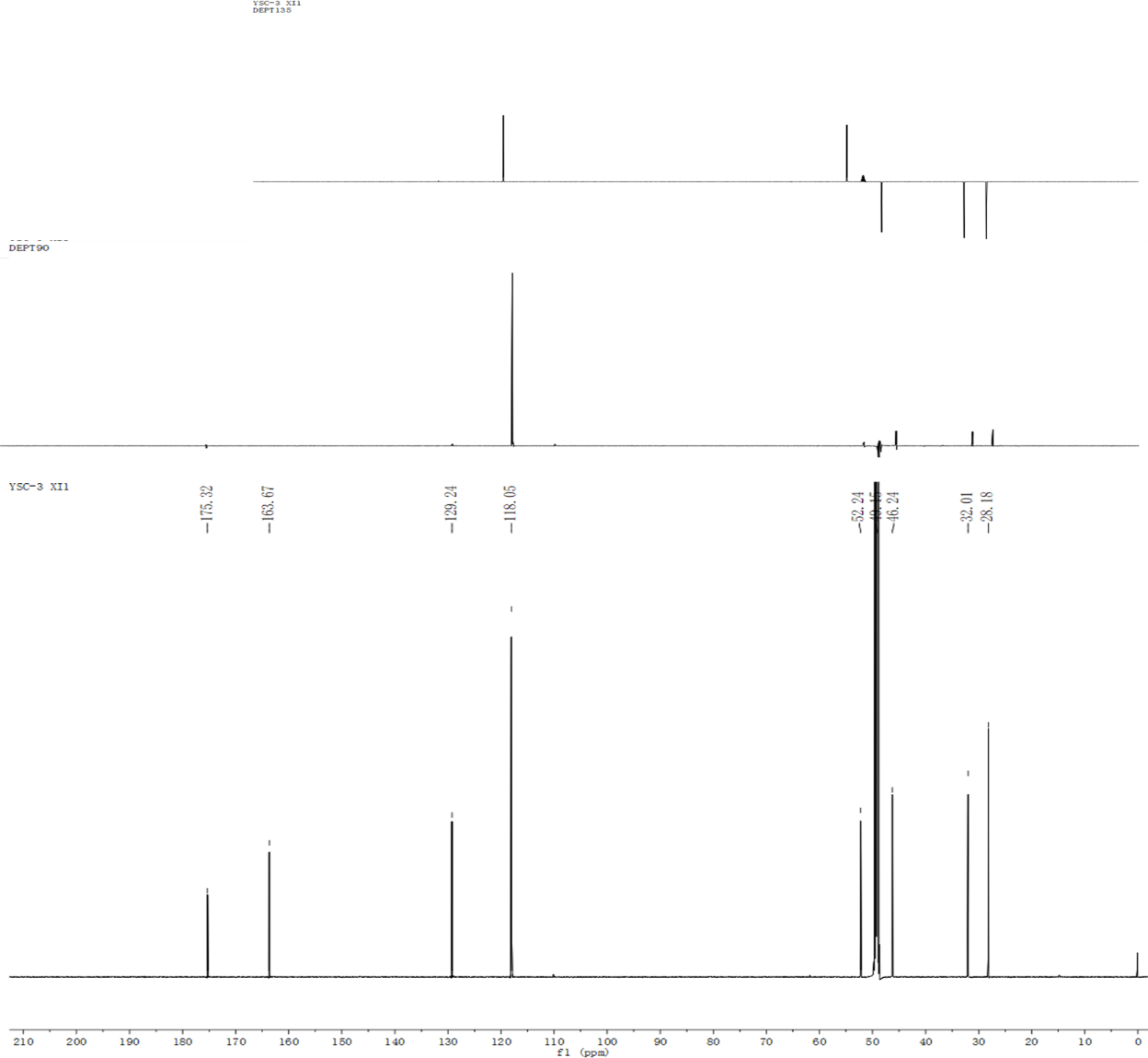


**Figure S3. ^13^C NMR spectrum of compound 1**


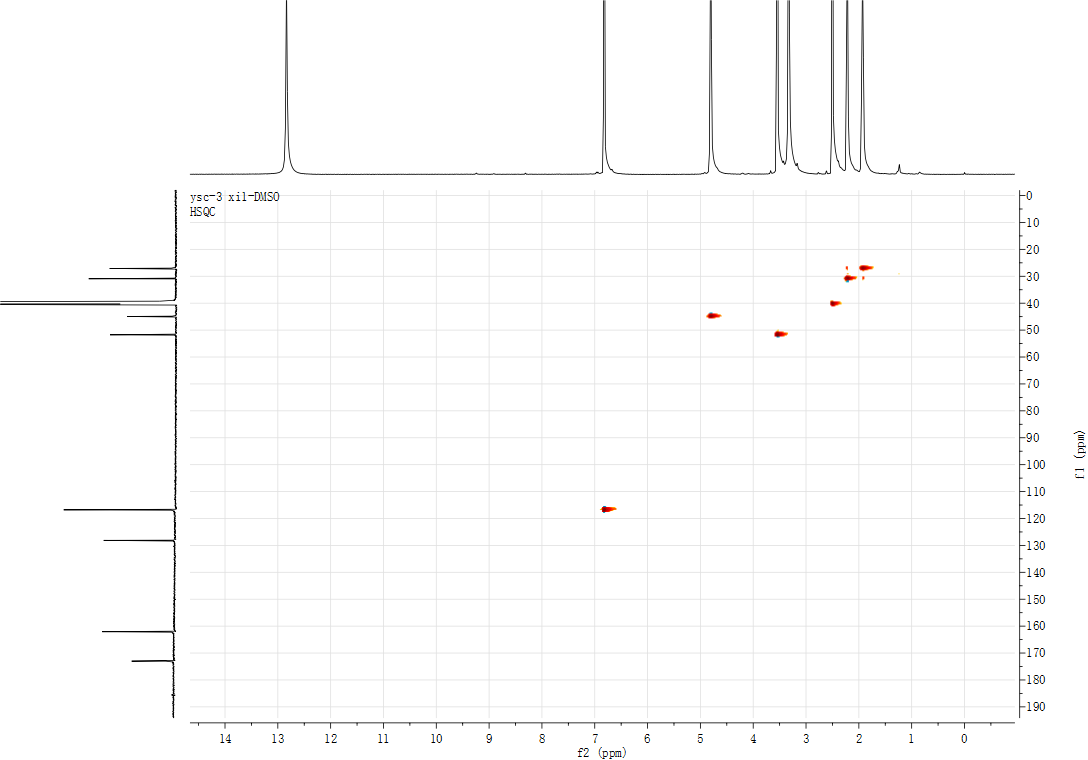


**Figure S4. HSQC spectrum of compound 1**


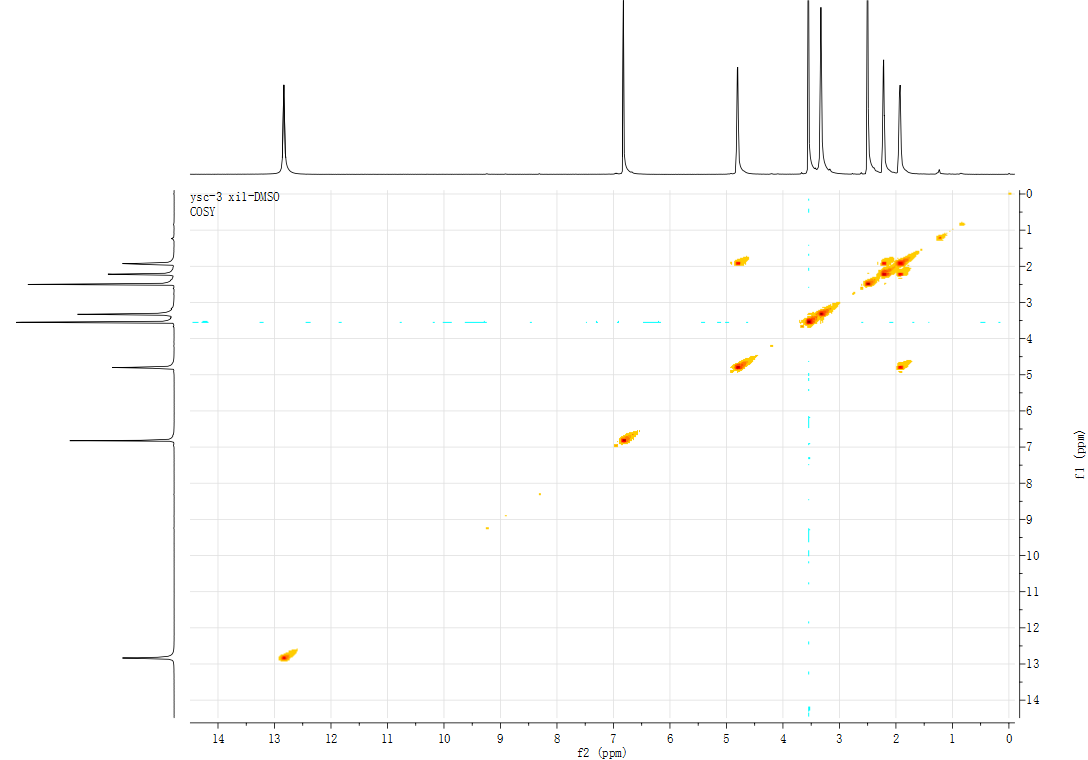


**Figure S5. ^1^H-^1^H COSY spectrum of compound 1**


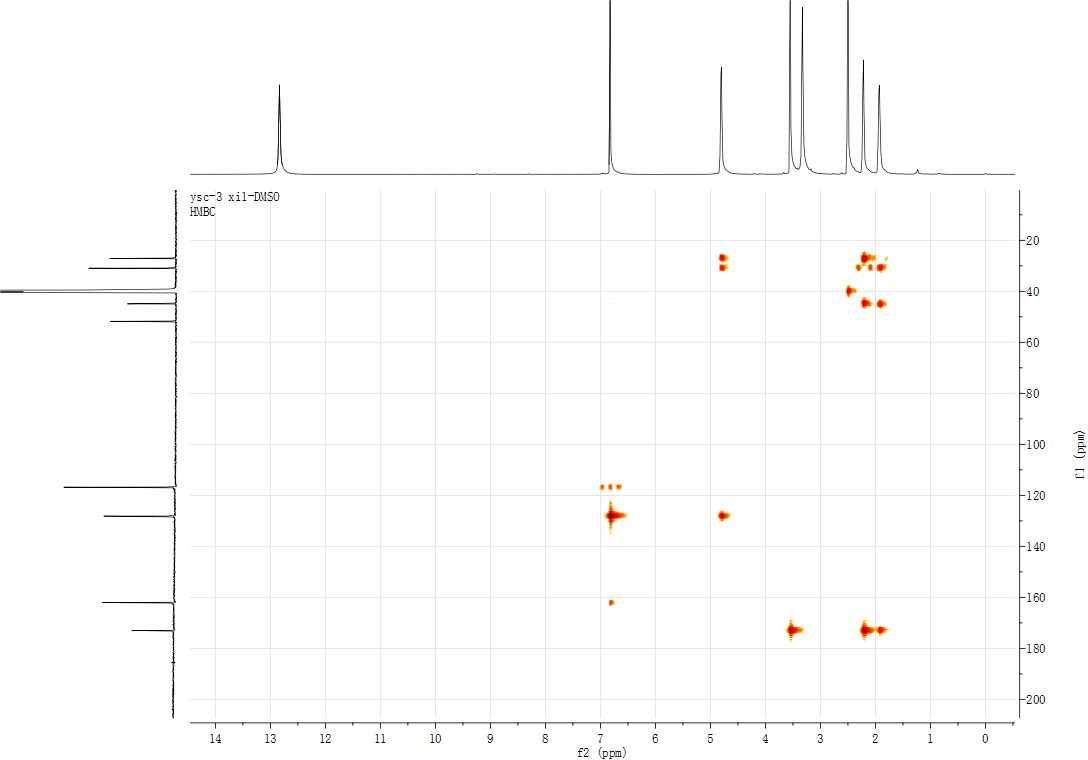


**Figure S6. HMBC spectrum of compound 1**


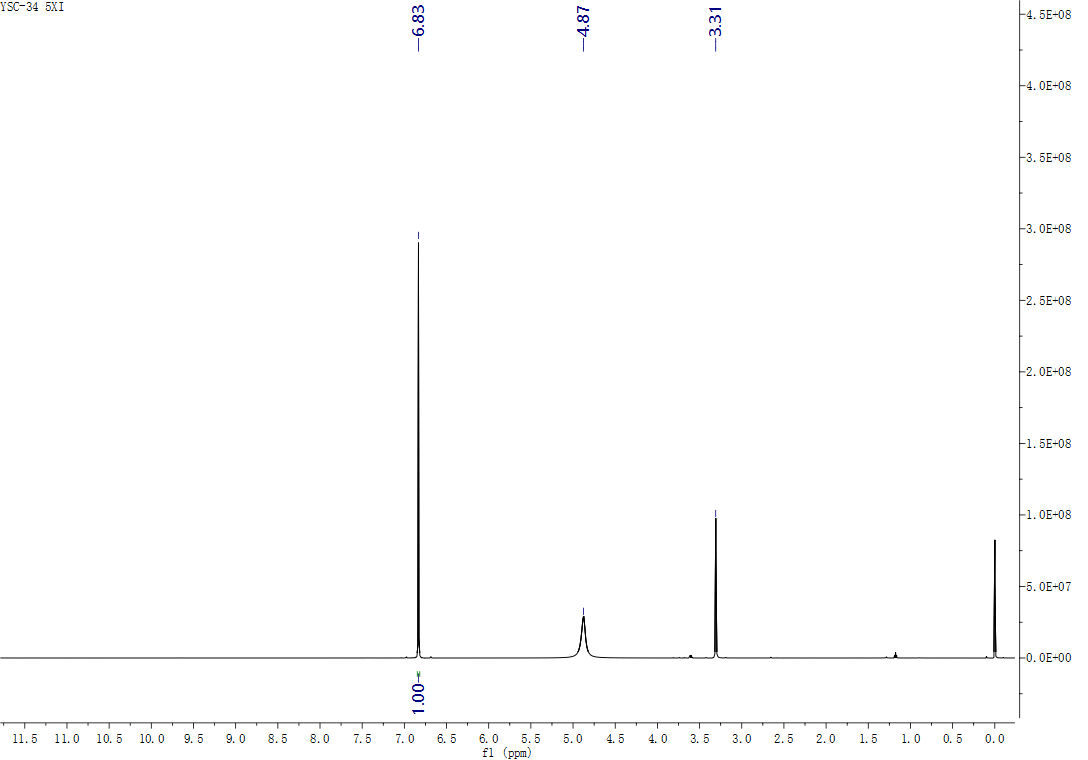


**Figure S7. ^1^H NMR spectrum of compound 2**


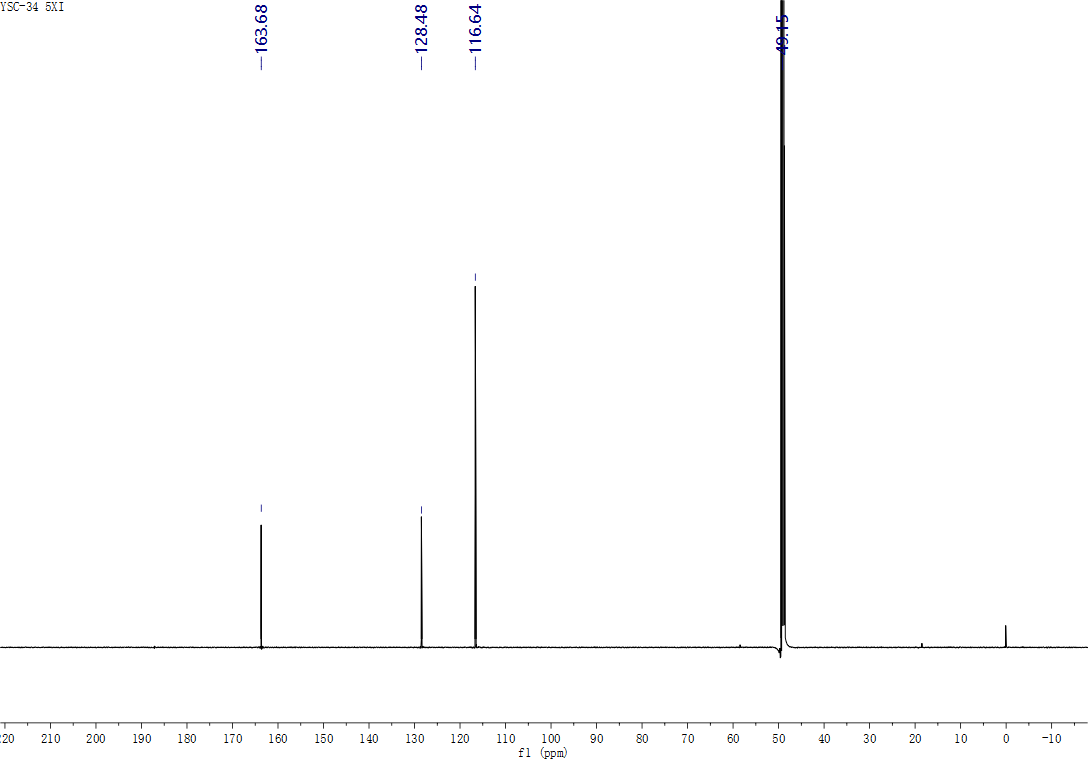


**Figure S8. ^13^C NMR spectrum of compound 2**


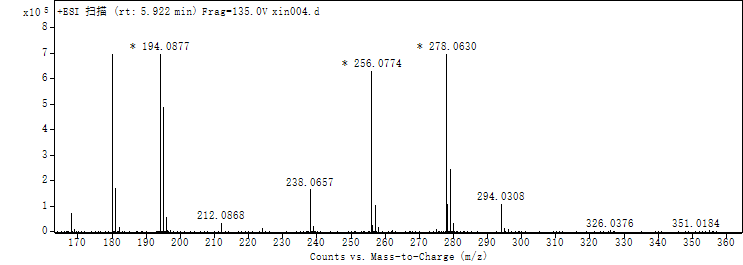


**Figure S9. HRESIMS of Compound 1**

**Figure S10. IR spectrum of compound 1**

**Figure S11.** Effect of **PAs** on cell cytotoxicity.

(Macrophages were incubated with various concentrations of **PAs** for 24 h, and cell viabilities were measured by MTT assay. The results are reported as a percentage compared to the untreated controls. **p＜0.05*, significant compared with **PAs**-uninfected RAW264.7)
